# From dialysis to transplantation: a 5-year longitudinal study on self-reported quality of life

**DOI:** 10.1186/1471-2369-15-191

**Published:** 2014-12-02

**Authors:** Nanna von der Lippe, Bård Waldum, Fredrik B Brekke, Amin AG Amro, Anna Varberg Reisæter, Ingrid Os

**Affiliations:** Institute of Clinical Medicine, University of Oslo, Oslo, Norway; Department of Nephrology Ullevål, Oslo University Hospital, Oslo, Norway; Department of Transplantation Medicine Rikshospitalet, Oslo University Hospital, Oslo, Norway

**Keywords:** Dialysis, Kidney transplantation, HRQOL, KDQOL-SF, Longitudinal, Clinical relevant change

## Abstract

**Background:**

Little is known how health related quality of life (HRQOL) change in the transition from dialysis to renal transplantation (RTX). Longitudinal data addressing the patient-related outcomes are scarce, and particularly data regarding kidney-specific HRQOL are lacking. Thus, the aim of the current study was to assess HRQOL in patients followed from dialysis to RTX. Furthermore, to compare HRQOL in RTX patients and the general population.

**Methods:**

In a prospective study, HRQOL was measured in a cohort of 110 patients (median age 53.5 (IQR 39–62) years, GFR 54 (45–72) ml/min/1.73 m^2^) in dialysis and after RTX using the self-administered Kidney Disease and Quality of Life Short Form version 1.3 (KDQOL-SF). Generic HRQOL in the RTX patients was compared to that of the general population (n = 5903) using the SF-36. Clinical important change after RTX was defined as difference in HRQOL of SD/2.

**Results:**

Follow-up time was 55 (IQR 50–59) months, and time after RTX was 41 (34–51) months. Four of nine domains in kidney-specific HRQOL improved after RTX, i.e. burden of kidney disease, effect of kidney disease, symptoms and work status. In SF-36, general health, vitality, social function and role physical improved after RTX, but none of the domains improved sufficiently to be regarded as clinically relevant change. There were highly significant differences in HRQOL between RTX patients and the general population after adjustment for age and gender for all items of SF-36 except for bodily pain and mental health.

**Conclusions:**

HRQOL improved in the transition from dialysis to transplantation, but clinical relevant change was only obtained in the kidney specific domains. HRQOL was perceived considerably poorer in RTX patients than in the general population. Our observations point to the need of improving HRQOL even after RTX, and should encourage further longitudinal research and clinical attention during treatment shift.

## Background

Patients with end stage renal disease (ESRD) dependent on dialysis have reduced quality of life not only compared to the general population [1–4], but also to patients with other chronic diseases [5, 6]. Kidney transplantation (RTX) is considered to be the optimal treatment for patients with ESRD with survival advantage compared to dialysis [7, 8]. Cross-sectional studies have indicated improvement in physical, mental and social aspects of health related quality of life (HRQOL) in patients after RTX compared to dialysis treatment [9, 10]. However, there is a scarcity of longitudinal studies addressing HRQOL during treatment shift from dialysis to RTX. The few studies suggest improved HRQOL, but most have a short follow-up time and a low number of patients [11–13]. The majority of the studies have used only generic HRQOL measures, and the limited numbers of studies using kidney-specific questionnaires included few patients with short follow-up [4, 14, 15]. Furthermore, perceptions of mental and physical aspects of HRQOL may change with time after transplantation [16–18]. We postulate that HRQOL will improve in the transition from dialysis to RTX, and that this applies not only for generic, but also for disease-specific issues.

Whether kidney transplanted patients will obtain the same HRQOL as that of the general population is unclear, as previous observations are inconsistent, with some reporting inferior [19–21], and others similar HRQOL scores in RTX patients and the general population [17, 22]. As renal transplanted patients may face new encounters such as side effects of a heavy drug regimen, infections and deteriorating graft function [23], we expect self-perceived HRQOL to be lower in RTX patients than in the general population.

Thus, the aims of the current longitudinal cohort study were to assess changes in HRQOL in the transition from dialysis to renal transplantation, and to compare HRQOL in transplanted patients with that of the general population.

## Methods

Between August 2005 and February 2007, a total of 301 patients from 10 different hospitals in Norway were included in a cross-sectional study. All patients >18 years receiving either hemodialysis (HD) or peritoneal dialysis (PD) for > two months were invited to participate [2]. Cognitive dysfunction, major psychiatric disorder and inadequate Norwegian language skills were exclusion criteria. Hospitalization during the investigation period led to exclusion, but patients could be enrolled four weeks or more after hospital discharge if they were clin0069cally stable. Close to one third of the prevalent dialysis population from both urban and rural areas were enrolled in the study. Study nurses and physicians had been trained in applying the study instruments to the patients. The questionnaires were answered during a regular HD session or during a scheduled visit for the PD patients. Further details regarding this study are given elsewhere [2].

**Figure 1:**
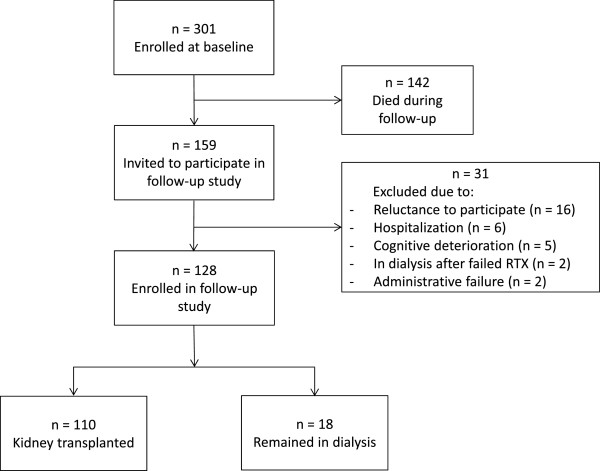
**Flowchart of enrolment.**

All included patients from the first study were invited to participate in the follow-up study, both patients who had been transplanted as well as patients who remained in dialysis (Figure 1). Inclusion and exclusion criteria were unchanged from the original study. The transplanted patients answered the questionnaires during a regular visit at the renal outpatient clinic. Clinical and demographic data were collected from hospital charts and/or direct questioning of the patients. Records of RTX were obtained from the Norwegian Renal Registry [24]. Comorbidity was assessed using the modified Charlson comorbidity index (CCI) [25]. The modified CCI is validated for dialysis patients [25] and kidney transplanted patients [26], and is a composite score of age and 17 weighted comorbid conditions including coronary artery disease, congestive heart failure, cerebrovascular disease, diabetes, malignancy and chronic pulmonary disease. In the current study all types of skin cancers, including basal cell carcinomas, were considered as tumors and given two points. Hepatitis without cirrhosis was regarded as mild liver disease. Diabetes as a comorbid condition scored one point, diabetes as cause of ESRD scored two points. CCI was also calculated without including age to evaluate the effect of age as a separate factor in statistical analyses. Data on HRQOL in the general population were collected in the Survey of Level of Living 2002 [27].

The Regional Committees for Medical and Health Research Ethics in Norway approved the protocols (2005/2010), and concession was obtained from the National Data Inspectorate. The study was accomplished according to the Helsinki Declaration [28]. Written and oral information were provided, and signed consent was obtained.

### HRQOL

The self-administered Kidney Disease and Quality of Life Short Form version 1.3 (KDQOL-SF) [29] consists of a kidney-specific and a generic part. The kidney-specific part contains 43 kidney-specific items that are summarized into 11 domains, i.e. symptoms, effect of kidney disease, burden of kidney disease, cognitive function, quality of social interaction, sleep, sexual function, social support, dialysis staff encouragement, patient satisfaction with care, as well as work status. The kidney-specific domains “dialysis staff encouragement” and “satisfaction with care” were not relevant in RTX patients, and thus excluded from the current analyses. One item, the overall health rating item, is not included in the 11 predefined domains of the KDQOL. Respondents rate their health on a 0 – 10 response scale ranging from worst possible to best possible health. The score was converted to a 0–100 score, where 100 was the best perceived overall health. The generic part of KDQOL-SF comprises the Medical Outcome Study 36-item Short Form Health Survey (SF-36) [30]. It consists of 36 items summarized into the eight conceptual domains physical function, limitation due to physical problems, bodily pain, general health, vitality, social function, limitation due to emotional problems, and mental health. Two component summary scores can be derived from the subscales, physical component summary score (PCS) and mental component summary score (MCS). Scores in KDQOL-SF were transformed into linear 0–100 point scores, with higher scores indicating better quality of life. KDQOL-SF has been validated for kidney transplanted patients and dialysis patients, also for use in longitudinal studies [31, 32].

Half a standard deviation (SD) of the baseline score in each domain was chosen as a measure of clinical relevant change in HRQOL [33]. This is equivalent to Cohen’s d = 0.50 [34].

### Statistical methods

Clinical and demographical data were presented either as mean ± SD if normally distributed, or as median with interquartile range (IQR) if data were skewed. Proportions were given for categorical variables. For normally distributed data, paired Student *t*-test was applied, while Wilcoxon signed rank tests were used if data were skewed. McNemar’s test was used for categorical data.

HRQOL scores were presented as mean ± SD despite being skewed. Data were presented in this manner in order to compare with other published HRQOL studies, however, non-parametric Wilcoxon signed rank tests were used as assumptions of normality were not fulfilled.

To evaluate whether change in HRQOL differed with respect to age, gender, transplant function, baseline dialysis modality, dialysis vintage, and time after RTX, stratified analyses were undertaken. Comparison of differences in delta values (change from dialysis to RTX) in all the KDQOL-SF domains in the cited strata were performed using the Student *t*- test as the delta values fulfilled the assumption of normality. Cut-off for age was set at the value ≥ 65 years and ≤ 45 years. These cut-offs were chosen to secure diversion in age and adequate sample sizes. Based on clinical experience and to ensure adequate sample sizes, the cut -off values for glomerular filtration rate (GFR), for time after RTX, and for dialysis vintage were set at 45 ml/min/1.73 m^2^, 36 months, and 12 months, respectively. Dialysis vintage was defined as the time from the first dialysis sessions until baseline.

Forward selection logistic regression models were built to identify predictors of clinical improvement of HRQOL after RTX compared to dialysis. Analyses were undertaken with the separate domains of KDQOL and SF-36 as dependent variables. The dependent variables were dichotomized based on the cut-off for clinical relevant change as described previously, i.e. delta values ≥ 0.5 SD. Gender, comorbidity, BMI, dialysis vintage and travel time to dialysis center were chosen as independent variables to assess predictors of improved HRQOL after RTX. The independent variables were categorized to fulfill the assumption of linearity of the logit. The cut-offs were set clinically at age ≥ 65 and ≤ 45 years, travel time to dialysis center ≥ 45 minutes, dialysis vintage ≥ 12 months, comorbidity ≥ 4 points and BMI ≥ 30 kg/m^2^.

Based on the mean reference values of the general population, expected mean scores were calculated, meaning the HRQOL scores that would be observed in the general population if they had equivalent age and gender distribution as the RTX patients [35]. Non-parametric one-sample test was used to compare the expected median scores with the crude median scores of the RTX group.

Missing values in SF-36 were substituted with the patient’s mean score if less than half of the subscales were missing. No substitution was done in KDQOL if a question was left open, the total score was calculated as suggested by the RAND group [36]. Missing data were treated by pair wise deletion in the statistical analyses.

All data were analysed using SPSS for Windows version 21 (IBM SPSS Statistics, New York, USA). Level of significance was set to p < 0.05.

## Results

From the original dialysis population of 301 patients, 110 patients had been transplanted, while 142 died during the 55 months of follow-up (Figure 1). The clinical and demographic characteristics of the transplanted patients are given in Table 1. Time since transplantation was 40.3 ± 14.4 months. Standard immunosuppressive regimen with cyclosporine or tacrolimus and mycophenolate mofetil (MMF) combined with (86/110) or without (3/110) prednisolone was used in the majority of the patients. Everolimus combined with MMF and prednisolone was used in 11/110 patients, and other different combinations in 10/110 patients.

**Table 1:** **Characteristics of the study population (n = 110) during dialysis and after renal transplantation**

	Dialysis	Transplanted	P value
Age	51.3 ± 14.8	56.6 ± 14.7	
Female gender, %	33.6		
Hemodialysis, %	75,5		
Time of follow-up, months		55.0 ± 6.5	
Time after RTX^1^, months		40.3 ± 14. 3	
Wait listed for RTX^1^ at baseline	36/110		
Time in dialysis, months	7 (3 – 15)	22 (15 – 36)^2^	
Glomerular filtration rate, mL/min/1.73 m^2^		54 (45 – 72)	
Systolic blood pressure, mmHg	141 ± 21	136 ± 15	0.03
Diastolic blood pressure, mmHg	80 ± 12	78 ± 10	0.10
Body mass index, kg/m^2^	25.9 ± 4.2	26.8 ± 5.3	0.012
Hemoglobin, g/dL	12.2 ± 1.5	13.4 ± 1.8	< 0.001
Albumin, mmol/L	39.3 ± 4.3	42.8 ± 3.5	< 0.001
C-reactive protein, mg/L	5 (2 – 9)	2 (1 – 7)	= 0.20
Cholesterol, mmol/L	4.4 ± 1.3	5.0 ± 1.2	= 0.001
Charlson Comorbidity Index	5 (3 – 6)	6 (4 – 7)	< 0.001
Diabetes (n)	19	25	= 0.11

Data are given as mean ± SD, median with IQR, or number. ^1^Renal transplantation; ^2^Time in dialysis at time of transplantation.

### Changes in HRQOL from dialysis to RTX

**Figure 2:**
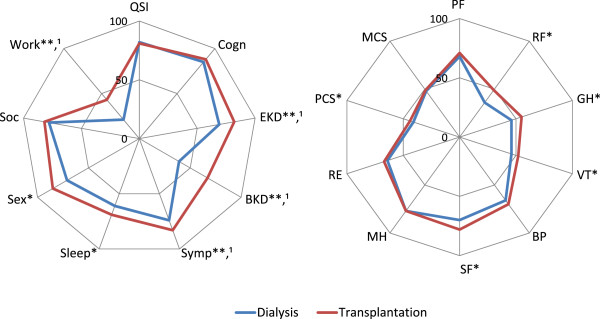
**Mean KDQOL-SF in 110 patients during dialysis and after transplantation.** *p < 0.05; **p < 0.001; ^1^Clinically relevant change: improvement > 0.5 SD. QSI: Quality social interaction; Cogn: Cognitive function; EKD: Effect of kidney disease; BKD: Burden of kidney disease; Symp: Symptoms; Sex: Sexual fuction; Soc: Social support; Work: Work status; PF: Physical function; RP: Role physical; GH: General health; VT: Vitality; BP: Bodily pain; SF: Social functioning; MH: Mental health; RE: Role emotional; PCS: Physical Component Summary Score; MCS: Mental Component Summary Score. Scores 0–100, higher number indicating better HRQOL.

Significant HRQOL improvements were seen after RTX in several of the kidney-specific HRQOL domains. Effect of kidney disease, burden of kidney disease, symptoms, sleep, sexual function and work improved, while cognitive function, social support and quality of social interaction did not (Figure 2). The kidney-specific “overall health” item improved after RTX, from 58 ± 20 to 68 ± 21 respectively, p < 0.001. In the generic domains, significant improvement was observed in limitation due to physical problems, vitality, general health and social function.

Changes in HRQOL scores were not perceived different between men and women, or between patients with low or high GFR in the stratified analyses. Neither did changes in HRQOL scores differ between dialysis modalities (peritoneal dialysis vs. hemodialysis). In age-stratified analyses, renal transplantation had different effect on younger patients than older patients in the domain quality of social interaction (Δ 8 ± 26 vs. Δ -5 ± 25, p = 0.035). In patients ≤ 45 years the score was 76 ± 22 in dialysis and 84 ± 14 after RTX, while in patients ≥ 65 years the score was 86 ± 16 in dialysis and 81 ± 19 after RTX.

Patients with the longest time in dialysis had a more pronounced change in the domain burden of kidney disease compared to patients with shorter time (Δ 42 ± 43 vs. Δ 21 ± 39, p = 0.016). Patients with dialysis vintage ≥ 12 months scored 33 ± 28 in dialysis and 75 ± 29 after RTX, while patients with dialysis vintage < 12 months scored 41 ± 25 and 63 ± 31.

Change in PCS differed between patients with shorter time after RTX compared with patients with longer time after RTX (Δ 8 ± 12 vs. Δ 1 ± 18, p = 0.045). Patients with shorter time after RTX had a score of 41 ± 9 in dialysis and 49 ± 10 at time of follow-up, and patients with longer time after RTX had a score of 41 ± 10 in dialysis and 42 ± 10 at time of follow-up. No association with time was observed in MCS.

### Clinical relevant change

The described clinical relevant improvement (half a SD) varied from 12 to 20 points for the different domains in KDQOL-SF, and 5 points for PCS and MCS. Using these thresholds, the domains effect of kidney disease, burden of kidney disease, work status, symptoms and the overall health item improved (Figure 2). None of the generic domains, including those with statistical significant changes, had clinical relevant change from dialysis to RTX, nor did any relevant clinical alterations occur in MCS and PCS (Figure 2).

The proportion of patients that reported improvement exceeding the cut-off for clinical relevant change ranged from 30 to 61% for the various KDQOL-SF domains. To identify predictors of the clinical relevant changes, separate logistic regression models were performed. Patients with CCI score ≥ 4 were more likely to perceive improved “general health” after RTX compared with patients with low comorbidity score (OR = 4.5, 95% CI 1.7 – 12.3, p = 0.003). BMI ≥ 30 kg/m^2^ in dialysis was associated with improvement in mental health after RTX (OR = 4.0, 95% CI 1.3 – 12.1, p = 0.016). For other domains in KDQOL-SF, no variables were identified that predicted improvement in kidney-specific or generic HRQOL after RTX.

### Comparison to the general population

**Figure 3:**
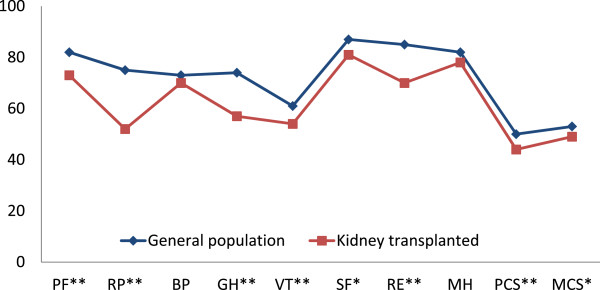
**Adjusted SF-36 mean values in kidney transplanted patients (n = 110) and the general population (n = 5903).** *p < 0.05; **p ≤ 0.001. PF: Physical function; RP: Role physical; GH: General health; VT: Vitality; BP: Bodily pain; SF: Social functioning; MH: Mental health; RE: Role emotional; PCS: Physical Component Summary Score; MCS: Mental Component Summary Score. Scores 0–100, higher number indicating better HRQOL. Mean scores adjusted for age and gender.

Overall, the crude SF-36 scores in RTX patients were statistical significantly lower than in the general population (n = 5903) in all domains except for bodily pain and mental health. The differences persisted after adjustment for age and gender (Figure 3). The most pronounced changes were found in physical function (9 points), limitations in physical role functioning (23 points), general health (17 points) and limitations in emotional role functioning (15 points).

## Discussion

An important novel observation in the present longitudinal study was the overall improvement in the kidney-specific HRQOL in transplanted patients. Kidney-specific data have so far been lacking or insufficient. Improvement would be expected shortly after transplantation, as the kidney function is retrieved. Our observation showed that this improvement was present after more than three years post-RTX, and specifically the perception of complaints related to kidney failure. The majority of the current RTX patients had mild to moderate chronic kidney disease, i.e. stage 2–3, and with fewer symptoms than would be expected with more advanced renal failure. The two domains, i.e. effect of kidney disease (EKD) and burden of kidney disease (BKD) had numerically the most pronounced improvement after RTX. BKD, which addresses how kidney disease may interfere with life, cause frustrations, and burdening the family, and EKD, being more related to daily-life restrictions and worries, were in the present study lower than what have been observed in a group of never-transplanted CKD (chronic kidney disease) patients with equivalent GFR [37]. In the latter study BKD scores were 85 compared to 67 in our study, while EKD scores were 92 and 82 in the never-transplanted CKD and the RTX patients, respectively. On the other hand, our observation of the HRQOL scores in the longitudinal cohort study was in accordance with the finding in a cross-sectional study of kidney transplanted patients with GFR in the same range, i.e. GFR 30–60 ml/min/1.73 m^2^, with unadjusted EKD scores 88 and BKD 78 [38]. These observations suggest that despite similar GFR, renal transplanted may actually fare poorer than non-transplanted patients. This assumption can only be corroborated by findings from cross-sectional studies as it is unlikely that we will get prospective longitudinal HRQOL studies including patients from CKD stage 2–3 and follow them for years until after RTX.

The KDQOL-SF has been assessed as a valid and reliable tool in RTX patients as well as in other CKD patients [32]. The internal consistency has been found to be particularly high for the domains EKD, BKD and symptoms in RTX patients [32], while some domains may be less relevant as they contain questions specifically for dialysis patients [29]. Previous experience with the use of KDQOL-SF is, however, limited in RTX patients. As far as we know, only two longitudinal studies using KDQOL-SF have been done, and with a small number of patients [14, 15]. One of the studies was published as a short abstract, and a small amount of data was revealed. Only 30 transplanted patients were included, all with kidney from living donors [15]. The other study included a highly selected small group of young transplanted patients attending a training program [14]. There are two cross-sectional studies that have used KDQOL and compared HRQOL in dialysis patients with that of RTX patients [39, 40]. While one of the studies was small and addressed ethnicity differences rather than treatment shift [40], the other large study had less than 35% response rate in the RTX group [39], which may lead to selection bias. The current study therefore may contribute noticeably to increased knowledge on kidney-specific HRQOL in the transition from dialysis to transplantation.

Patients over 65 years of age actually perceived poorer HRQOL in the domain “quality of social interaction” in the conversion from dialysis to RTX, while younger patients perceived improvement. One possible explanation to this finding could be that older patients may become more socially isolated after RTX than younger. Older patients may have less personal contacts, a smaller network of family and friends and thus participate more infrequent in social activities than younger [41, 42]. Social interaction experienced during dialysis sessions should perhaps not be underestimated.

Dialysis vintage affected the change in the domain burden of kidney disease during follow-up, with a larger improvement in patients with the longest time in dialysis. This may not be surprising, as the patients with longest dialysis vintage may have felt more burdened, and therefore perceived that RTX relieved the frustrations and interference more than in those with shortest dialysis vintage. Divergent results regarding associations between dialysis vintage and HRQOL after RTX are reported, and only for generic domains, i.e. physical domains [18, 43, 44].

One item which has gained little attention in previous studies is the question “Overall, how would you rate your health” in the kidney-specific part of the questionnaire. As far as we know, only one study has published data on this question [39], and the authors surprisingly found the score to be lower in transplanted than in dialysis patients, with no apparent explanation. In contrast to that finding, the current study showed substantially improvement after RTX. This is in accordance with our clinical experience with RTX patients, and actually confirms that beyond the survival benefit, patient-related outcome is enhanced after RTX.

The changes in the generic HRQOL domains were less impressive than the changes observed in the disease-specific domains. The present study actually contradicted previous reports that generic HRQOL is perceived substantial higher in RTX than in dialysis patients [22, 45]. However, the majority of previous studies are cross-sectional, and therefore selection bias cannot be excluded. Furthermore, adjustments for other variables were rarely undertaken in these studies. When comparing dialysis patients on the waiting list with renal transplanted patients, the difference in HRQOL was less pronounced and quite comparable to our study [20]. A possible explanation for the difference in HRQOL in the different studies could be that the majority of patients in the cross-sectional studies would never be considered for RTX due to comorbidity or high age [22, 45]. Moreover, the only previous longitudinal study (n = 20) using KDQOL-SF [14] actually reported change in generic HRQOL from dialysis to transplantation in the same range as the present study.

We cannot rule out the possibility of a time effect contributing to the discrepancies of findings in our study and in some previous studies, as those studies comparing HRQOL changes from dialysis to transplantation have been obtained in the early post-transplantation period [4, 11, 17]. Less is known about HRQOL after the initial “honeymoon” period in the transplanted patients. In the present study, PCS was perceived lower in patients who had been transplanted for more than three years compared to those with shorter time, but no such difference was found for the kidney-specific items. It has been suggested that HRQOL in kidney transplanted patients might follow a U-shaped pattern with the highest score the first year and then after five years [46], but this time effect needs to be further explored.

Statistical significant difference in HRQOL may not necessarily be perceived as clinical important change for the patient. Clinically meaningful changes in HRQOL may vary with the disease, level of severity, gender, cultural background, socioeconomic status and other factors [47]. Furthermore, one cannot rule out that the greater change in the most impaired patients could actually reflect a statistical phenomenon, the regression to the mean, rather than a clinical alteration. Determining clinically meaningful change has been a matter of substantial debate, and several methods, either anchor-based or distribution-based, have been proposed to define and calculate this dimension, as described by Crosby et al. in a comprehensive review [47]. Anchor-based methods compares changes with a reference, an anchor, which can be objective measures such as response to treatment, or subjective measures such as patient-reported global ratings of change in health status. Distribution-based methods are based on statistical properties of the scale and include effect sizes. One of the most common used distribution-based tools is Cohen’s d, where the effect size (ES) is calculated from the change in mean scores divided by the standard deviation [34]. Cohen’s guidelines suggests that ES of 0.2 - 0.5 reflect a small effect, 0.5 – 0.8 a moderate effect and > 0.8 a large effect. There are no established thresholds for clinical important changes for the KDQOL-SF questionnaire, neither for dialysis nor kidney transplanted patients. In the current study, we chose pragmatically the distribution-based method of half a standard deviation [33] to define the cut-off clinical relevant change. This is equivalent to Cohen’s d = 0.50. To evaluate whether the method used in the current study represents the “true” threshold for these patients, more studies are needed, also combining anchor-based and distribution based methods.

Most patients in dialysis may expect their lives to change dramatically after transplantation, and thus overestimate the benefits of the transplantation [48]. Actually, the generic HRQOL scores in the current study were overall much lower in the RTX patients then in the general population for all domains in SF-36 except for bodily pain and mental health. Our finding is in keeping with a previous Norwegian study of young transplant recipients [49], and with observations in other patients with chronic diseases [50, 51]. The reduced HRQOL in RTX patients compared to the general population may reflect that these patients although transplanted still have a chronic kidney disease, often associated comorbidity, and also side effects of the medication, anxiety of rejection, malignancy, and infections.

The longitudinal design, the fairly large sample size and the fact that none of the patients were lost to follow-up strengthen the present study. Data quality was good with less than 2% missing data in SF-36. The transplantation recruitment procedure was similar in all dialysis units, and transplantations took place in one center. Some particular aspects may limit the generalizability of our results as very few patients were non-Caucasian, and the time in dialysis before RTX was fairly short. The inclusion criteria may have led to selection bias towards healthier patients as patients with cognitive dysfunction, drug abuse or hospitalized patients were excluded. although they were considered representative of the Norwegian dialysis population [2], Evaluation of response shift was not addressed when designing the study. This is a phenomenon suggesting that the self-perceived perceptions of HRQOL may be incited by changes in health, e.g. transplantation, which may then lead to changes in internal standards of measurement, priorities or in the conceptualization of perceived HRQOL [52].

## Conclusion

While the kidney-specific HRQOL improved statistically and clinically in the transition from dialysis to transplantation, the changes in generic HRQOL items were not large enough to be regarded as clinically important change. Furthermore, RTX patients did not obtain HRQOL similar to that of the general population. We suggest that patient-related outcomes such as kidney-specific and generic HRQOL should be used routinely in RTX patients in the clinical setting, and not only for research purpose. This will provide important information about the well-being of these patients. Health professionals should be aware of the reduced HRQOL in patients with ESRD, which may persist even after RTX, and focus on how HRQOL could be improved. We recommend that not only should HRQOL, including kidney-specific items, be measured in patients with CKD 4–5, but also annually after the initiation of dialysis. Repeated measurements should also be undertaken after the conversion to RTX. Future clinical trials with longitudinal design and repeated measurements of HRQOL are required to better describe the trajectory after renal transplantation.

## Abbreviations

RTX: Renal transplantation
KDQOL-SF: Kidney disease and quality of life short form version 1.3
HRQOL: Health related quality of life
SF-36: Medical outcome study 36-item short form health survey
KDQOL: Kidney disease quality of life
PCS: Physical component summary score
MCS: Mental component summary score
BKD: Burden of kidney disease
EKD: Effect of kidney disease
CCI: Charlson comorbidity index
BMI: Body mass index
GFR: Glomerular filtration rate
ESRD: End stage renal disease
HD: Hemodialysis
PD: Peritoneal dialysis
CKD: Chronic kidney disease.
